# Targeting the endogenous cannabinoid system to treat neuropathic pain

**DOI:** 10.3389/fphar.2014.00028

**Published:** 2014-03-03

**Authors:** Benjamin K. Lau, Christopher W. Vaughan

**Affiliations:** Pain Management Research Institute, Kolling Institute of Medical Research, Northern Clinical School, University of SydneySydney, NSW, Australia

**Keywords:** neuropathic pain, endocannabinoid, 2-arachidnoylglycerol, anandamide, FAAH, MAGL

Chronic neuropathic pain is a debilitating condition that remains poorly treated by current medications. Preclinical studies have indicated that cannabinoid receptor agonists have analgesic efficacy in neuropathic pain models, but this is accompanied by undesirable side effects. In recent years, novel strategies targeting the endogenous cannabinoid system have emerged, which are being mooted as safer alternatives. A recent clinical trial, however, has demonstrated that a new endocannabinoid modulator is ineffective against osteoarthritic pain, despite exhibiting efficacy during the preclinical stage. Further basic and clinical work is needed to resolve this disparity.

## Cannabinoids and chronic pain

Chronic pain is a prevalent and costly health care problem (Torrance et al., 2006). A particularly persistent, severe and debilitating form of chronic pain is neuropathic pain. This syndrome manifests following damage or dysfunction of the peripheral nerves, spinal cord, or brain; or can be caused by stroke, multiple sclerosis or diabetes. Unfortunately, the currently recommended pharmacological treatments for neuropathic pain display poor efficacy and illicit undesirable side effects (Dworkin et al., 2010).

There is growing clinical evidence indicating that the cannabis constituent, Δ^9^-tetrahydrocannabinol (THC), and synthetic cannabinoid agonists have efficacy in chronic pain states (Lynch and Campbell, 2011). These human studies are based upon substantial preclinical evidence. A wide range of animal models of neuropathic pain have demonstrated that cannabinoid agonists reverse the common symptoms of neuropathic pain, including allodynia (to cool and innocuous mechanical stimuli) and hyperalgesia (to noxious thermal and mechanical stimuli) (Fox et al., 2001; Scott et al., 2004). Unfortunately, this therapeutic intervention is also associated with a number of adverse effects, including sedation and motor/cognitive impairment. Having said this, the therapeutic window between the desired and adverse effects of cannabinoids has not been systematically examined. Thus, it remains to be determined whether cannabinoids can produce pain relief at doses below the side effect threshold.

## The endogenous cannabinoid system—a target for pain relief

Since exogenous cannabinoid agonists act globally throughout the central nervous system to produce their effects, isolation of the desired therapeutic action from the unwanted side effects has remained a difficult challenge. To overcome this problem, alternative approaches at targeting cannabinoid signaling have been explored (Petrosino and Di Marzo, 2010; Piscitelli and Di Marzo, 2012). Unlike exogenous cannabinoids, endogenous ligands of the cannabinoid system are synthesized in an “on demand” fashion. This suggests specific, localized release of these transmitters only in regions where their actions are pertinent. Thus, targeting endocannabinoids may provide a more effective strategy in relieving pain devoid of side effects.

Endocannabinoids are present in multiple pain-modulating regions throughout the CNS, including the periaqueductal gray (PAG), rostral ventral medulla (RVM) and spinal cord dorsal horn, where their levels are enhanced by acute nociceptive stimuli and stress (e.g., Walker et al., 1999; Hohmann et al., 2005). Interestingly, endocannabinoid levels within these regions are also enhanced in chronic pain models (Jhaveri et al., 2007; Petrosino et al., 2007; Guindon et al., 2013). Exogenous administration of AEA or 2-AG produces THC-like effects, but each elicits a distinct subset of the total effects observed with exogenous cannabinoid administration. Specifically, in animal models, AEA administration has been reported to produce antinociception alone (Cravatt et al., 2001), or evoke the full tetrad of cannabinoid agonist effects—antinociception, hypothermia, hypolocomotion and catalepsy (Smith et al., 1994). By contrast, 2-AG administration evokes only a subset of these effects (Lichtman et al., 2002). While major endocannabinoids, such as N-arachidonoyl ethanolamide (anandamide or AEA) and 2-arachidonoyl glycerol (2-AG), act via cannabinoid CB1 and CB2 receptors in a manner similar to THC and synthetic cannabinoid agonists, they may also modulate nociception via non-cannabinoid receptor targets. This is particularly the case for anandamide, which has actions on transient receptor potential vanilloid 1 (TRPV1) and peroxisome proliferator-activated receptor-α (PPAR-α), both of which are cellular targets implicated in nociception (Jhaveri et al., 2007; Di Marzo and De Petrocellis, 2012).

The actions of endocannabinoids are tightly regulated by enzymatic degradation. In particular, AEA is degraded via fatty acid amide hydrolase (FAAH), and 2-AG via monoacylglycerol lipase (MAGL) (Cravatt et al., 1996; Dinh et al., 2002). In addition, two serine hydrolases, ABHD6 and ABHD12, have recently been implicated in the hydrolysis of 2-AG (Blankman et al., 2007; Savinainen et al., 2012); however, their presence in pain pathways has yet to be confirmed. In recent years, a number of pharmacological tools have been developed which selectively inhibit FAAH and MAGL (Kathuria et al., 2003; Long et al., 2009a; Ahn et al., 2011). Inhibition of these degradative enzymes is thought to specifically enhance endocannabinoids where they are produced on demand, resulting in more localized receptor activation compared to globally acting exogenous agonists. Considerable attention has been focused on developing degradation inhibitors to indirectly target the endocannabinoid system.

## Novel endocannabinoid modulators—FAAH and MAGL inhibitors

A number of groups have demonstrated that selective FAAH inhibitors, such as URB597, OL-135, PF-3845, PF- 04457845 and ST-4070 alleviate the mechanical and cold allodynia induced in a range of animal models of chronic pain (Russo et al., 2007; Kinsey et al., 2009, 2010; Ahn et al., 2011; Caprioli et al., 2012; Guindon et al., 2013). Although it has not been examined systematically, this analgesic effect occurs at doses that do not elicit the tetrad of cannabinoid-induced side effects. The evidence in support of FAAH inhibitors, however, is not unanimous. For example, our group originally found that systemic administration of URB597 had no effect on mechanical allodynia in the partial nerve ligation (PNL) model of neuropathic pain (Jayamanne et al., 2006), despite the synthetic analog of THC, HU-210 abolishing allodynia in the same study.

The contrasting findings between these studies might be due to a number of factors. Firstly, the above studies examined a range of different neuropathic pain models. Indeed, we have observed that, unlike the PNL model, URB597 reduces allodynia (albeit at higher doses) in the sciatic nerve chronic constriction injury (CCI) model (unpublished data). Secondly, the wide range of different FAAH inhibitor compounds administered at varying doses may account for the contrasting efficacy on allodynia, particularly since some of compounds in question did not display clear dose dependence in pain assays (e.g., Ahn et al., 2011). Thirdly, depending on the acute or chronic pain assay examined, it is possible that endocannabinoid activation of CB1 and TRPV1 receptors may have opposing effects on nociception, thus confounding the observed results (Maione et al., 2006). Together, these studies suggest that FAAH inhibitors may be efficacious for neuropathic pain, but more systematic studies need to be conducted to confirm their therapeutic potential.

Compared to FAAH inhibitors, there have been relatively fewer studies examining MAGL inhibitors in pain models. This is mainly due to their later identification and development. Nevertheless, systemic administration of the MAGL inhibitors, JZL-184 and KML-29, have been shown to alleviate allodynia in certain neuropathic pain models, without eliciting the full tetrad of cannabinoid-induced effects (Kinsey et al., 2009, 2010, 2013; Schlosburg et al., 2010; Guindon et al., 2013; Ignatowska-Jankowska et al., 2013). Interestingly, while analgesic efficacy is maintained during chronic administration of low doses of JZL184, tolerance has been shown to develop at higher doses (Kinsey et al., 2013). This latter observation is clinically relevant, since conditions like chronic pain often require long-term drug treatment.

## Therapeutic potential of endocannabinoid modulators

In a recent clinical trial, the FAAH inhibitor, PF-04457845 was found ineffective in patients with osteoarthritic pain (Huggins et al., 2012). This was despite a clear abolition of FAAH activity and elevation in plasma AEA levels in almost all subjects tested. This finding directly contrasts those observed by the same group in an animal model of inflammatory and non-inflammatory pain, in which PF-04457845 produced a potent antinociceptive effect (Ahn et al., 2011). While the reason for this contrasting efficacy between humans and animals is not clear, it should be noted that the former study was likely confounded by a number of human factors (Di Marzo, 2012). Furthermore, the study examined only an osteoarthritic model of pain. Thus, the utility of FAAH inhibitors in the treatment of neuropathic pain remains to be explored.

## Synergistic/antagonistic interactions of endocannabinoids

In chronic pain states, patients often receive drugs in combination. The aim of this approach is to produce greater or potentially synergistic analgesia at lower drug doses. Indeed, there is some clinical evidence in favour of the combined use of cannabinoid and opioid agonists in chronic pain patients, although synergy has yet to be established (Abrams et al., 2011).

This combinatorial approach might also be applied to the endocannabinoid modulators described above. One promising idea would be to inhibit both FAAH and MAGL (Pertwee, 2013). In this regard, dual inhibitors of both FAAH and MAGL have recently been developed (Long et al., 2009b; Ramesh et al., 2013), although they remain to be systematically tested in chronic pain models. Because FAAH and MAGL act via distinct pathways to degrade AEA and 2-AG, dual inhibition of these degradative enzymes should boost levels of both endocannabinoids. It is unknown whether this will produce an additive or synergistic effect. Therefore, it would be interesting to observe the effect of dual FAAH/MAGL inhibition in neuropathic pain models, particularly in terms of their efficacy and therapeutic window relative to FAAH or MAGL inhibition.

It should be noted that whilst the antinociceptive effect of MAGL inhibitors is specifically abolished by CB1 receptor antagonism or knockout (Kinsey et al., 2009, 2010), the effect by FAAH inhibitors is not only abolished/reduced by CB1 and CB2 receptor blockade (Russo et al., 2007; Kinsey et al., 2009, 2010), but also antagonism of TRPV1 receptors and PPAR-α (Caprioli et al., 2012). Since TRPV1 and PPAR-α are predominantly pro-nociceptive targets, this suggests FAAH inhibition may produce competing, opposing actions on antinociception. Thus, it would be interesting to examine the effect of a FAAH inhibitor in combination with a TRPV1 antagonist. In this regard, the endocannabinoid-related agent, N-arachidonoyl-serotonin is a dual FAAH/TRPV1 inhibitor found to be highly effective in neuropathic pain models (De Novellis et al., 2011). In addition to pro-nociceptive targets, FAAH inhibition has been demonstrated to produce pro-nociceptive metabolites by diverting AEA breakdown through other metabolic pathways, such as cyclooxygenase-2 (COX-2) (Gatta et al., 2012). Hence, another potential interaction worth examining is dual inhibition of FAAH and COX-2. Such an approach may also lead to enhanced analgesic efficacy (Fowler et al., 2009).

Together, the above studies importantly highlight that while some endocannabinoid pathways act synergistically, others may act antagonistically to reduce antinociception. Therefore, prizing apart these subcellular pathways may provide a means to further improve the safety and efficacy of endocannabinoid modulators.

## Future directions

While the lack of efficacy of a FAAH inhibitor in a recent clinical trial is disappointing, there are alternative approaches targeting the endocannabinoid system which may still be of promise. These need to be first addressed in animal studies before proceeding with further clinical trials. In particular, the efficacy of the highly specific, individual MAGL inhibitors and the recently developed, dual FAAH/MAGL inhibitors need to be explored in neuropathic pain models. Furthermore, the use of these degradation inhibitors in combination with agents that act on related targets should be investigated (Di Marzo, 2012). Both drug efficacy and side-effect profiles need to be examined systematically. This may eventually lead to an effective pharmacotherapy to treat the problematic condition of neuropathic pain.
